# Maternal Inflammation During Pregnancy and Cord Blood Metabolomic Signatures in the Context of HIV Exposure

**DOI:** 10.3390/metabo15120765

**Published:** 2025-11-25

**Authors:** Tianyue Fu, Ellen C. Francis, Carolyn Kinkade, Rhoda S. Sperling, Yunping Qiu, Irwin J. Kurland, Jennifer Jao, Stephanie Shiau

**Affiliations:** 1 Department of Biostatistics and Epidemiology, Rutgers School of Public Health, Piscataway, NJ 08854, USA; tf274@scarletmail.rutgers.edu (T.F.); ellen.francis@rutgers.edu (E.C.F.); caw136@sph.rutgers.edu (C.K.); 2 Department of Obstetrics, Gynecology, and Reproductive Health, Icahn School of Medicine at Mount Sinai, New York, NY 10029, USA; rhoda.sperling@mssm.edu; 3 Department of Medicine, Division of Endocrinology, Fleischer Institute for Diabetes and Metabolism, Albert Einstein College of Medicine, Bronx, NY 10461, USA; yunping.qiu@einsteinmed.edu (Y.Q.); irwin.kurland@einsteinmed.edu (I.J.K.); 4 Department of Pediatrics, Department of Medicine, Northwestern University Feinberg School of Medicine, Chicago, IL 60611, USA; jjao@luriechildrens.org

**Keywords:** pregnancy, HIV exposure, metabolomics, lipidomics, inflammation

## Abstract

**Background/Objectives**: Pregnant people with HIV (PWH) are more likely to experience systemic inflammation than pregnant people without HIV (PWoH), which may contribute to adverse outcomes in HIV-exposed uninfected (HEU) infants; however, the underlying mechanisms are not well studied. This study examined associations between maternal inflammatory markers during pregnancy and cord blood inflammatory markers and metabolomic signatures. **Methods**: Between 2011 and 2025, pregnant PWH and PWoH were enrolled at 24–28 weeks of gestational age. Maternal plasma was analyzed for inflammatory markers [interleukin (IL)-6, high-sensitivity C-reactive protein (hsCRP), soluble TNF-α receptor 1 (sTNFR1) and 2 (sTNFR2), soluble CD163 (sCD163), soluble CD14 (sCD14)]. At delivery, cord blood was collected for measurement of IL-6, TNF-α, IFN-γ, and IL-10 and for targeted metabolomics by ultra-performance liquid chromatography–mass spectrometry. Spearman correlation, linear regression, and weighted correlation network analysis (WGCNA) were used to evaluate associations, stratified by HIV exposure. **Results**: This study included 22 PWH and 47 PWoH and their infants. Among HEU infants, but not HUU infants, maternal IL-6 correlated with cord blood TNFα (r = 0.443, *p* < 0.05) and maternal sTNFR1 correlated with both cord blood TNFα (r = 0.617, *p* < 0.05) and IFNγ (r = −0.517, *p* < 0.05). WGCNA identified five metabolomic modules. In the HEU group, naternal sCD14 was positively associated with a metabolomic module characterized by lysophosphotidylecholines in the HEU group. **Conclusions**: We identified distinct patterns in the relationships between maternal inflammation and infant immune–metabolic profiles by HIV exposure status. These findings suggest that HIV infection, even with viral suppression, may alter the maternal–fetal inflammatory interface and influence early metabolic programming.

## 1. Introduction

Pregnant people living with HIV (PWH) experience heightened inflammation and immune activation compared to pregnant people without HIV (PWoH) during pregnancy [1]. Of particular concern is the elevated level of soluble CD14 (sCD14), a marker of monocyte/macrophage activation closely linked to HIV-associated gut barrier dysfunction and microbial translocation [2]. In non-pregnant PWH receiving antiretroviral therapy (ART), elevated inflammatory markers, such as interleukin-6 (IL-6), high-sensitivity C-reactive protein (CRP), and sCD14, have been associated with increased morbidity and mortality [3,4]. These markers may therefore serve as important indicators of HIV-related comorbidity risk.

An inflammatory maternal environment during pregnancy may contribute to elevated inflammation in the infant, potentially leading to adverse outcomes, such as preterm birth, low birth weight [5], or neurodevelopmental delays [6], altered immune function [7], and impaired metabolic health [8,9,10]. Patterns of immune dysregulation have been identified in HIV-exposed uninfected (HEU) infants, which may underlie their increased vulnerability to adverse outcomes [7]. Additionally, HIV-exposed infected infants have been shown to exhibit elevated levels of inflammation and monocyte activation [8].

The induction and resolution of inflammation are complex metabolic processes, with lipid signaling having a key role in maintaining metabolic homeostasis and immunity [11]. In contrast to single molecules, such as glucose, lipids are composed of a variety of distinct molecules depending on their headgroup, backbone, and acyl chain. Prior studies have identified dysregulated metabolic and immune pathways in HEU infants, including disruptions in lipid metabolism and elevated proinflammatory immune mediators [12]. Maternal inflammation triggered by HIV infection, even when controlled with ART, may reprogram metabolic and immunological pathways in utero. Metabolomics and lipidomics offer powerful tools for identifying clusters of metabolites and lipid subspecies that may be altered in HEU infants. However, limited data exist on the relationship between maternal inflammation during pregnancy and the infant cord blood metabolome in the context of HIV exposure.

In this study, we examine associations between maternal inflammation during pregnancy and cord blood inflammatory and metabolomic profiles among HEU and HIV-unexposed uninfected (HUU) infants. We speculate that maternal inflammation is associated with offspring inflammation and metabolomic profiles. Understanding how in utero exposure to maternal inflammation shapes infant immune and metabolic pathways may identify targets for future interventions to improve outcomes for HEU infants.

## 2. Materials and Methods

### 2.1. Study Population

This study used data from a cohort of pregnant PWH and PWoH and their infants, enrolled between 2011 and 2015 at the Mount Sinai Hospital ambulatory obstetrics–gynecology practice between 24 and 28 weeks of gestational age (GA). The clinic provides high-risk obstetric care to pregnant PWH and routine prenatal care to PWoH from the same geographic catchment area. Pregnant PWoH were recruited from the midwifery clinic, which serves individuals with uncomplicated pregnancies. Pregnancies with multiple gestations, those ending in spontaneous/therapeutic abortions, or intra-uterine fetal demise (IUFD), and those resulting in an infant with HIV infection were excluded. All participants provided written informed consent. This study was approved by the Institutional Review Board of the Icahn School of Medicine at Mount Sinai and Rutgers University.

### 2.2. Maternal Markers of Inflammation and Immune Activation

Plasma interleukin 6 (IL-6), high-sensitivity C-reactive protein (hsCRP), soluble TNF-α receptor 1 (sTNFR1) and 2 (sTNFR2), soluble CD163 (sCD163), and soluble CD14 (sCD14) were measured using enzyme immunoassay techniques according to the manufacturer’s directions from samples collected at 24–28 weeks of gestation. Assays were performed in duplicate at the Special Infectious Diseases Laboratory of the Ann and Robert H. Lurie Children’s Hospital of Chicago, and the mean of two replicates was used for downstream analysis. Standard dilution procedures were performed for samples with initial values outside the limit of detection (LOD) until readings were within range, and results were adjusted by the dilution factor.

### 2.3. Cord Blood Markers of Inflammation

Cord blood IL-6, TNF-α, interferon (IFN)-γ, and interleukin 10 (IL-10) were measured using Milliplex^®^ (MilliporeSigma, Burlington, MA, USA) MAP multiplex assays with Luminex^®^ (Luminex Corporation, Austin, TX, USA) instrumentation. Each sample was assayed in duplicate, and all samples were tested from the same subject on the same plate. The samples were stored in a freezer at −80 °C from the time of collection to the time of sample testing, for an average of two years [13].

### 2.4. Cord Blood Metabolomics and Lipidomics

Metabolomics and lipidomics were measured in a sub-group of participants with available specimens (N = 50, 17 HEU, 33 HUU). Widely targeted small metabolite (WTSM) screening (600+ small polar metabolites), widely targeted lipidomic (WTL) profiling (1300+ lipid species in 26 lipid classes), and widely targeted eicosanoid (WTE) screening (120+ eicosanoids) were performed using umbilical cord blood plasma samples. Small metabolites were extracted in 80% methanol/water, and lipids in 90% ethanol/water, and eicosanoids using a solid-phase extraction (SPE) column, eluted in 100% methanol, dried, and reconstituted in a loading buffer. Analyses were conducted at the Albert Einstein College of Medicine Stable Isotope and Metabolomics Core Facility using Waters Aquity^®^ Ultra Performance Liquid Chromatography coupled with an ABSciex^®^ 6500+ QTrap Mass Spectrometer (UPLC-MS) in Multiple Reaction Monitoring (MRM) mode with a gradient appropriate for each column. An ACE^®^ pentafluorophenyl (PFP) column was used for WTSM assay, a Waters Charged Surface Hybrid (CSH) Fluorophenyl column for the WTL assay, and a Waters BEH Shield RP18 column for the WTE assays. MultiQuant^®^ (ABSciex) software version 3.0.3 was used for data processing, including metabolite identification and quantification relative to internal standards.

### 2.5. HIV Status, Demographics, and Clinical Information

Newborns were classified as HEU vs. HUU as determined by maternal serological HIV ELISA testing and ART history per self-report and medical and pharmacy-record review. Demographics and clinical information were obtained from clinical charts, interviews, and physical examinations collected at visits as per study protocols. Birth weight-for-age z (WAZ) and length-for-age z (LAZ) were calculated from United States (U.S.) growth standards [14].

### 2.6. Statistical Analysis

Descriptive characteristics of the pregnant participants and their newborns were summarized using means and standard deviations, medians and interquartile ranges, or counts and percentages, as appropriate. Group comparisons were conducted using *t*-tests, Wilcoxon rank-sum tests, chi-squared tests, or Fisher exact tests. Maternal inflammatory marker and cord blood inflammatory marker concentrations were natural log-transformed to approximate a normal distribution and compared between groups using Wilcoxon rank-sum tests.

Our analytical workflow proceeded in several steps. First, we assessed relationships between maternal and cord blood inflammatory markers using Pearson correlation coefficients, stratified by HIV exposure status (HEU vs. HUU). A correlation heatmap was constructed using the corrplot package in R [15], displaying absolute Pearson correlation coefficients greater than 0.2 at a type I error of 0.05.

Second, we conducted a metabolomic network analysis including only the dyads with complete maternal and cord blood data (N = 50, 17 HEU, 33 HUU). We applied weighted correlation network analysis (WGCNA) to generate a global metabolomic network, identify module-based metabolomic signatures, and define key compounds within each signature [16]. Details on the WGCNA algorithm, R package (version 1.73), and nomenclature have been reported elsewhere [17]. For network creation, WGCNA parameters were set according to published recommendations [17,18], with a minimum module size of 30 compounds, module dissimilarity threshold ≥ 0.20, and scale-free fit index R^2^ > 0.80. The first eigenvector of each module represented the corresponding metabolomic signature. Key compounds (top 10) for each module were identified by ranking absolute Pearson correlations with the eigen vector. These were used to assign descriptive labels to the network modules.

Third, we used partial Pearson correlations to assess the association between the maternal inflammatory markers and the cord blood metabolomic eigenvectors, stratified by HIV exposure status and adjusted for maternal age, infant sex, and birth weight-for-age Z-score. Lastly, for modules significantly associated with maternal inflammatory markers (*p*-value < 0.05), we conducted linear regression analyses with the top 10 compounds to determine which metabolites contributed most strongly to the overall associations.

All statistical analyses were performed using SAS^®^ 9.4 (Cary, NC, USA) and R Statistical Software (4.4.0).

## 3. Results

### 3.1. Characteristics

A total of 22 pregnant PWH and 47 PWoH and their infants were included in the analysis (Table 1). Pregnant PWH were older than the PWoH (mean age 29.0 vs. 24.2 years, *p* = 0.002). No differences in maternal race/ethnicity, education level, employment status, family history of diabetes, substance use in pregnancy, pre-pregnancy BMI, or gestational diabetes were noted. Among the pregnant PWH, 11 (50%) had a CD4 cell count at enrollment > 350 cells/mm^3^ and 20 (90.9%) had an HIV RNA level < 100 copies/mL at delivery. All but two pregnant PWH received ART during pregnancy.

Several maternal inflammatory markers measured during pregnancy differed between groups. Pregnant PWH had higher median IL-6, sTNFR1, and sCD163 levels compared to pregnant PWoH. HEU infants were more likely to be delivered via cesarean section than the HUU infants (72.73 vs. 8.51%, *p* < 0.01). No differences between groups were observed in the rates of preterm birth, small-for-gestational (SGA) outcome, or birth anthropometrics (Table 1). Additionally, there were no significant differences in cord blood inflammatory cytokines between groups.

### 3.2. Relationships Between Maternal Inflammatory Markers and Infant Cord Blood Inflammatory Markers

**Figure 1:**
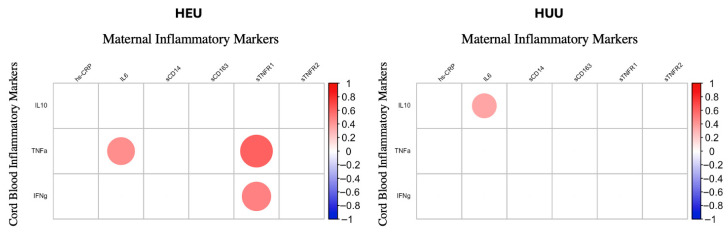
**Relationships between maternal inflammatory markers and cord blood inflammatory markers by HIV exposure status.** The heat map displays Spearman correlations (where rho is >0.2) between maternal inflammatory markers and cord blood metabolic-related cytokines by HIV exposure status. Circle color corresponds to correlation value. Circle size corresponds to *p*-value. A larger circle indicates a smaller *p*-value. Abbreviations—HEU: HIV-exposed uninfected; HUU: HIV-unexposed uninfected; IL: interleukin; hs-CRP: high-sensitivity C-reactive protein; sTNFR: soluble TNF-α receptor; sCD14: soluble CD14; sCD163: soluble CD163; TNFα: tumor necrosis factor alpha; IFN-γ: interferon gamma.

The relationships between maternal and cord blood inflammatory markers differed by HIV exposure status (Figure 1). Among the HEU infants, maternal IL-6 was significantly positively associated with cord blood TNFα (r = 0.443, *p* < 0.05), and maternal sTNFR1 was positively associated with cord blood TNFα (r = 0.617, *p* < 0.05) and with IFN*γ* (r = −0.517, *p* < 0.05). In contrast, among the HUU infants, maternal IL-6 showed a significant positive association with cord blood IL-10 (r = 0.351, *p* < 0.05).

### 3.3. Relationships Between Maternal Inflammatory Markers and Cord Blood Metabolome/Lipidome by HIV Exposure Status

**Figure 2:**
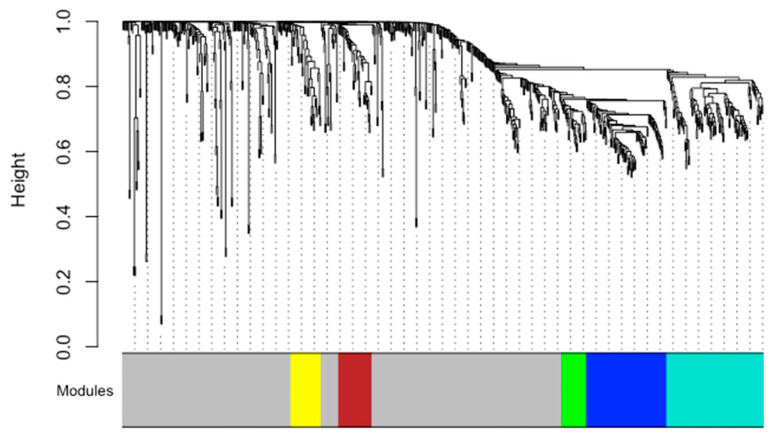
**Metabolite networks represented by clusters of correlated metabolites.** A total of six networks was constructed from 726 metabolites using the weighted correlation network analysis approach. The constituents of each metabolite network are presented. Note: YELLOW module/lysophospholipids and cholesteryl ester module (**LP-CE module**) (34 metabolites and lipids): primarily composed of lysophosphatidylcholine (LPC) (n = 12, 35.3%), lysophosphatidylethanolamines (LPEs) (n = 9, 26.5%), phosphatidylcholines (PCs) (n = 9, 26.5%), and cholesteryl esters (CEs) (n = 4, 11.7%). BROWN module/phosphatidylethanolamine module (**PE module**) (38 metabolites and lipids): primarily composed of phosphatidylethanolamine (PE) (n = 27, 71.1%), phosphatidylcholine (PC) (n = 1, 2.6%), phosphatidylserine (PS) (n = 2, 5.2%), anionic lipid phosphatidic acid (PA) (n = 4, 10.5%), and other (n = 4, 10.5%). GREEN module/polyunsaturated triacylglycerol (TAG) module (**PUFA module**): primarily composed of unsaturated TAG (n = 26, 92.9%) and saturated TAG (n = 2, 7.1%). BLUE module/saturated and unsaturated TAG module (**TAG module**) (91 metabolites and lipids) (28 metabolites and lipids): primarily composed of saturated triacylglycerols (TAGs) (n = 69, 75.8%) and unsaturated TAGs (n = 22, 24.2%). TURQUOISE module/saturated and monosaturated TAG module (**SM-TAG module**) (108 metabolites and lipids): primarily composed of saturated TAGs (96, 88.9%), a few unsaturated TAGs (n = 10, 9.3%), and diacylglycerols (DAGs) (n = 2, 1.8%). GREY is ungrouped compounds (**Ungrouped**) (427 metabolites and lipids): metabolites and lipids that failed to form a network.

Supplementary Material Tables S1 and S2 include all metabolites and their module loadings. The global metabolomic network analysis identified five distinct modules (Figure 2). These included:

(1) **Yellow module/lysophospholipid and cholesteryl ester module** (**LP-CE module**)—comprised primarily of lysophospholipids (LPCs, LPEs), phosphatidylcholines (PCs), and cholesteryl esters (CEs)

(2) **Brown module/phosphatidylethanolamine module** (**PE module**)—comprised mainly of diacly PEs and ether linkages (plasmalogens)

(3) **Green module/polyunsaturated triacylglycerol (TAG) module** (**PUFA module**)—comprised largely of polyunsaturated TAGs with long-chain fatty acid tails

(4) **Blue module** (**TAG module**)—contained various TAG species with medium fatty acid tails and some with palmitic or myristic acid tails

(5) **Turquoise module/saturated and monosaturated TAG module** (**SM-TAG module**)—comprised predominately of saturated and monosaturated TAGs with long-chain fatty acid tails and diacylglycerols (DAGs).

The grey module (Ungrouped) included compounds that did not cluster into a module and was not considered further.

**Figure 3:**
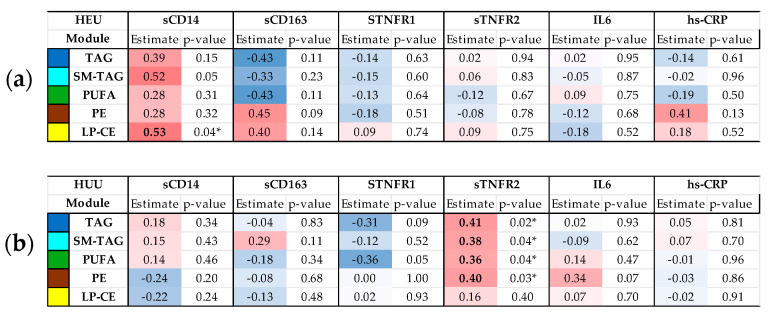
**Partial Pearson correlation coefficients between maternal markers of inflammation and immune activation and cord blood metabolite/lipid networks adjusted for maternal age and fetal sex.** (**a**) Association for HEU (HIV-exposed uninfected). (**b**) Association for HUU (HIV-unexposed uninfected). Darker red indicates a stronger positive correlation; darker blue indicates a stronger negative correlation. Bold *p*-value < 0.05. Abbreviations—HEU: HIV-exposed uninfected; HUU: HIV-unexposed uninfected; IL: interleukin; hs-CRP: high-sensitivity C-reactive protein; IFN-γ: interferon gamma; LP-CE: lysophospholipid and cholesteryl ester; PE: phosphatidylethanolamine; PUFA: polyunsaturated triacylglycerol; TNFR: soluble TNF-α receptor; sCD14: soluble CD14; sCD163: soluble CD163; TNFα: tumor necrosis factor alpha; TAG: triacylglycerol, SM-TAG: saturated and monosaturated TAG. * *p*-value < 0.05.

Partial correlations between maternal inflammatory markers and cord blood metabolomic signatures differed by HIV exposure status (Figure 3). Among the HEU infants, maternal sCD14 was positively correlated with the cord blood LP-CE module. In contrast, among the HUU infants, maternal sTNFR2 showed positive correlations with all cord blood metabolomic modules except the LP-CE module.

From the metabolomic signatures associated with maternal inflammatory markers, several individual lipid species that characterized these signatures were also significantly associated with maternal inflammatory markers (Table 2). Among HEU, higher maternal sCD14 levels were associated with higher concentrations of several LPCs and one LPE species. Among HUU, higher maternal sTNFR2 levels were positively associated with select lipid species from the PE, PUFA, SM-TAG, and TAG signatures.

## 4. Discussion

In this study of pregnant PWH and PWoH in New York City, we identified distinct HIV-related differences in the association between maternal inflammation during pregnancy and cord blood inflammatory markers. We observed that the pattern of associations between maternal inflammatory markers and cord blood metabolomic signatures differed by HIV exposure status. These findings suggest that maternal HIV infection, even in the context of viral suppression, may alter the maternal–fetal inflammatory interface and influence fetal metabolic programming in HIV-specific ways.

Consistent with previous studies [19,20,21], we observed differential patterns of inflammatory and immune activation markers in pregnant PWH compared to pregnant PWoH. In the HEU group, maternal sTNFR1 and maternal IL-6 were positively correlated with cord blood TNF*α*, and maternal sTNFR1 was also correlated with IFN*γ*. By contrast, among HUU infants, maternal IL-6 was correlated with cord blood IL-10. These findings align with prior reports demonstrating maternal–infant immune correlations in the context of HIV, supporting the hypothesis that maternal inflammation may contribute to a heightened inflammatory profile in HEU infants [22]. However, not all studies have observed such associations; for example, a study conducted in Tanzania reported no correlation between maternal inflammation and cord blood cytokines [23]. Differences in population characteristics (e.g., pre-pregnancy BMI, anemic status), timing of sample collection, or environmental exposures could explain these discrepancies.

Our results suggest that maternal inflammation may represent one mechanism through which HIV affects the fetal immune environment. IL-6 is a key proinflammatory cytokine involved in acute-phase responses [24], and has been shown to cross the placenta or stimulate placental cytokine production [25]. Persistent maternal inflammation may prime the fetal immune system toward a proinflammatory phenotype, potentially contributing to the infectious morbidity and developmental challenges reported among HEU infants.

Through WGCNA, we identified five distinct metabolomic and lipidomic signatures in cord blood. These modules represented complex lipid classes differentiated by their headgroups, backbones, and fatty acid linkages, including TAGs, DAGs, LPCs, PEs, and conjugated FAs. Lipid structural diversity influences a variety of metabolic functions, including energy storage, intra- and extracellular signaling, and the induction and resolution of acute and chronic inflammation [11]. For example, LPCs are the bioactive derivatives of PCs that can modulate the release of proinflammatory factors. However, the association between plasma concentrations and different disease states has been inconsistent, with higher levels observed in some metabolic and inflammatory conditions and lower levels observed in certain cancers and infectious states [26]. TAGs with shorter carbon chains (e.g., 41–52 carbons) and fewer double bonds (e.g., 0–4) tend to be composed of saturated (14:0, 16:0, 18:0) and monounsaturated (18:0) fatty acids [27]. Saturated fatty acids can potentiate inflammatory responses through activation of toll-like receptors (TLRs) [28].

The primary difference in the cord blood metabolome pattern between HEU and HUU neonates appeared to reflect differences in inflammatory markers levels between PWH and PWoH. Pregnant PWH had higher levels of monocyte and macrophage activation markers, and higher maternal, sCD14 was associated with the LP-CE metabolomic module among HEU infants. In contrast, PWoH had higher levels of sTNFR2, and higher maternal sTNFR2 was associated with lipid species across several cord blood metabolome modules in HUU infants.

Among HEU infants, higher maternal sCD14 levels were associated with higher cord blood concentrations of several LPC species including LPC (16:0), LPC (18:1), LPC (18:2), LPC (20:4), and LPE (18:1). LPC metabolites play critical roles in neurodevelopment, particularly in neuronal myelination and brain maturation. In addition, LPCs also serve as critical transporters for fatty acids and docosahexaenoic acid (DHA 22:6) to the fetal brain. For example, evidence in humans shows that when the transport protein for LPC is compromised, microcephaly and hypomyelination can result [29]. Among HUU infants, higher maternal sTNFR2 was associated with higher levels of key lipid species in the PE, PUFA, SM-TAG, and TAG modules. sTNFR1 and sTNFR2 are the primary receptors for TNFα and have distinct signaling pathways [30]. sTMFR1 is generally associated with mediating proinflammatory activation, whereas sTNFR2 has been linked to cell survival, tissue repair, and neuroprotection [31,32]. The positive association between sTNFR2 and beneficial PUFA species in HUU infants—particularly arachidonic acid (AA 20:4), alpha-linolenic acid (ALA 18:3), and linoleic acid (LA 18:2)—may reflect differences in maternal diet or supplement use. These PUFAs are essential for fetal neurodevelopment [33,34], and their fetal availability depends on maternal intake and other maternal-placental factors, including choline status and transporter expression [33]. As such, ensuring optional nutritional support for PWH during pregnancy may help mitigate the subtle neurocognitive differences observed among HEU children [35].

To our knowledge, this is the first study to examine associations between maternal inflammation during pregnancy and neonatal cord blood metabolomic signatures by HIV exposure status. Our findings suggest that maternal immune activation may influence specific lipid pathways relevant to fetal development and that these relationships differ between HEU and HUU infants. These results highlight potential biological mechanisms contributing to adverse outcomes observed among HEU infants, including altered neurodevelopment and metabolic health.

This study has several limitations worth noting. The sample size was relatively small, especially for stratified analyses, which may limit statistical power. For metabolomics analyses, fewer HEU than HUU participants were available, which may bias our results toward significant associations in the HUU group. Additionally, there may be residual confounding from unmeasured factors (e.g., maternal diet, microbiome composition, ART regimen). Despite these limitations, our findings generate novel hypotheses regarding the interplay between maternal inflammation, fetal metabolism, and long-term infant health, particularly in the context of perinatal HIV exposure.

## 5. Conclusions

In summary, our study identified a heightened inflammatory milieu among pregnant PWH and revealed distinct associations between maternal inflammation and neonatal cord blood metabolomic signatures, particularly among HEU infants. Using WGCNA, we characterized metabolomic and lipidomic networks in cord blood and found that sCD14, a marker of monocyte/macrophage activation, was positively associated with key metabolomic signature in HEU infants. This signature, enriched in lysophosphotidylecholines, comprised compounds that play critical roles in fatty acid transport and neurodevelopment. The observed associations suggest that maternal inflammation may influence fetal metabolic programming in ways that could contribute to adverse outcomes among HEU infants. Further research is needed to replicate these findings in larger cohorts and to elucidate the mechanistic pathways and long-term consequences of these maternal–fetal interactions. Such work may inform future strategies aimed at improving health outcomes for HEU children.

## Figures and Tables

**Table 1:** Characteristics of pregnant people and infants included in the sample (N = 69).

Maternal Characteristics		PWoH (N = 47)	PWH (N = 22)	*p*-Value
**Age (years)**	Mean (SD)	24.2 (4.71)	29.0 (5.84)	<0.001
**Race/ethnicity**	N (%)			
White		1 (2.13)	1 (4.55)	0.75
Black/African American		19 (40.4)	9 (40.9)	
Hispanic		23 (48.9)	9 (40.9)	
Other		4 (8.52)	3 (13.6)	
**Highest education level**	N (%)			
Some high school or less		7 (14.9)	2 (9.09)	0.80
High school diploma or equivalent		16 (34.0)	8 (36.4)	
Some college or higher		24 (51.1)	12 (54.6)	
**Employed**	N (%)	16 (34.0)	7 (31.8)	0.86
**Family history of diabetes**	N (%)	12 (57.1)	5 (38.5)	0.29
Missing		26	9	
**Illicit substance or alcohol use in pregnancy**	N (%)	0 (0.0)	1 (4.76)	0.31
Missing		0	1	
**Tobacco use in pregnancy**	N (%)	0 (0.0)	1 (5.0)	0.30
Missing		0	2	
**Pre-pregnancy BMI (kg/m^2^)**	Mean (SD)	26.4 (5.49)	27.5 (7.63)	0.56
**CD4 cell count at enrollment** > **350 cells/mm^3^**	N (%)		11 (50)	--
**HIV RNA level < 100 copies/mL at delivery**	N (%)	--	20 (90.9)	--
**Antiretroviral therapy during pregnancy**	N (%)			--
No ART		--	2 (9.09)	
NNRTI-based ^1^			6 (27.3)	
PI-based ^2^			11 (50.0)	
INSTI-based ^3^			2 (9.09)	
>3 classes of antiretrovirals			1 (4.6)	
**Maternal markers of inflammation and immune activation**				
IL-6 (pg/mL)	Median (IQR)	1.51 (0.84, 6.75)	0.73 (0.49, 1.82)	0.017
hs-CRP (ng/mL)	Median (IQR)	2922 (1212, 28,283)	6003 (2724, 12,582)	0.26
sTNFR1 (ng/mL)	Median (IQR)	2.08 (1.42, 2.54)	1.52 (1.19, 1.90)	0.03
sTNFR2 (ng/mL)	Median (IQR)	5.05 (2.36, 17.99)	16.78 (4.71, 43.82)	0.07
sCD14 (ng/mL)	Median (IQR)	1808 (1604, 1939)	1525 (1353, 1703)	0.08
sCD163 (ng/mL)	Median (IQR)	528 (428, 765)	459 (343, 584)	0.02
**Infant characteristics**		**HUU (N = 47)**	**HEU (N = 22)**	
Preterm (<37 weeks gestational age)	N (%)	2 (4.26)	2 (9.09)	0.59
Low birthweight (<1500 g)	N (%)	3 (6.38)	4 (18.2)	0.20
Small-for-gestational age	N (%)	3 (6.38)	4 (18.18)	0.20
C-section delivery	N (%)	4 (8.51)	16 (72.73)	<0.001
Birth weight-for-age Z-score	Median (IQR)	−0.19 (−0.82, 0.40)	−0.57 (−1.12, −0.16)	0.054
Birth length-for-age Z-score	Median (IQR)	0.089 (−0.46, 0.70)	−0.251 (−0.82, 0.46)	0.16
**Infant cord blood markers of inflammation**				
IL-6 (pg/mL)	Median (IQR)	13.74 (13.74, 35.87)	14.59 (13.74, 44.70)	0.77
TNFα (pg/mL)	Median (IQR)	10.91 (8.67,14.73)	14.01 (10.18,16.78)	0.54
IFN-γ (pg/mL)	Median (IQR)	0.60 (0.60, 0.61)	0.60 (0.60, 0.61)	0.07
Missing		1	2	
IL-10 (pg/mL)	Median (IQR)	1.67 (0.36, 3.03)	1.84 (0.82, 3.97)	0.95
Missing		1	3	

*p*-values for continuous variables from *t*-tests or Wilcoxon tests, and for categorical variables from chi-square or Fisher exact tests, as appropriate. No participants had gestational diabetes. ^1^ Nevirapine- or Rilpivirine-based. ^2^ Lopinavir/ritonavir-, Atazanavir-ritonavir-, or Darunavir/ritonavir-based. ^3^ Raltegravir-, Elvitegravir-, or Dolutegravir-based. Abbreviations—PWH: people living with HIV; PWoH: people without HIV; HEU: HIV-exposed uninfected; HUU: HIV-unexposed uninfected; IQR: interquartile range; SD: standard deviation; BMI: body mass index; NNRTI: non-nucleoside reverse transcriptase inhibitor; PI: protease inhibitor; INSTI: integrase inhibitor; IL: interleukin; hs-CRP: high-sensitivity C-reactive protein; sTNFR: soluble TNF-α receptor; sCD14: soluble CD14; sCD163: soluble CD163; TNFα: tumor necrosis factor alpha; IFN-γ: interferon gamma.

**Table 2:** Results from individual linear regression models for the top 10 compounds for each cord blood metabolomics module that was associated with maternal inflammatory markers, by HIV exposure status.

HEU			HUU											
Maternal sCD14			Maternal sTNFR2											
Cord Blood LP-CE Module	β	*p*-Value	Cord Blood PE Module	β	*p*-Value	Cord Blood PUFA Module	β	*p*-Value	Cord Blood SM-TAG Module	β	*p*-Value	Cord Blood TAG Module	β	*p*-Value
LPC (16:0)	2.70	0.012 *	PE(18:1/18:1)	0.30	0.010 *	TAG54:5-FA16:0	0.26	0.034 *	TAG50:0-FA18:0	0.32	0.010 *	TAG54:5-FA16:0	0.30	0.009 **
LPC (18:1)	2.60	0.017 *	PE(P-18:1/20:4)	0.23	0.046 *	TAG54:6-FA20:4	0.25	0.043 *	TAG50:1-FA18:1	0.31	0.013 *	TAG54:6-FA20:4	0.28	0.012 *
LPC (20:4)	2.47	0.011 *	PE(P-18:0/20:4)	0.24	0.16	TAG56:6-FA22:4	0.25	0.047 *	TAG48:1-FA18:1	0.30	0.016 *	TAG56:7-FA22:5	0.28	0.016 *
LPC (18:2)	2.36	0.029 *	PE(O-18:0/20:4)	0.22	0.06	TAG56:6-FA18:1	0.24	0.047 *	TAG48:1-FA14:0	0.30	0.018 *	TAG56:6-FA18:2	0.27	0.019 *
LPE (18:1)	2.34	0.022 *	PE(P-16:0/20:4)	0.19	0.08	TAG56:7-FA18:2	0.24	0.045 *	TAG54:3-FA16:0	0.28	0.022 *	TAG56:7-FA20:4	0.26	0.021 *
LPC (20:3)	2.19	0.07	PE(O-16:0/22:4)	0.18	0.13	TAG56:7-FA20:4	0.24	0.06	TAG50:2-FA16:0	0.27	0.031 *	TAG56:6-FA18:1	0.25	0.034 *
LPE (18:2)	2.09	0.05	PE(P-16:0/22:5)	0.16	0.11	TAG56:6-FA18:2	0.24	0.05	TAG54:3-FA20:2	0.27	0.030 *	TAG56:6-FA16:0	0.25	0.032 *
LPC (20:2)	2.05	0.05	PE(P-18:0/18:1)	0.14	0.25	TAG56:7-FA22:5	0.23	0.06	TAG53:2-FA16:0	0.24	0.048 *	TAG56:7-FA22:4	0.23	0.050 *
LPE (20:3)	1.73	0.16	PE(P-16:0/18:1)	0.14	0.27	TAG56:6-FA16:0	0.23	0.07	TAG52:2-FA16:0	0.24	0.05	TAG56:7-FA18:2	0.22	0.06
LPE (20:4)	1.65	0.13	PE(P-18:0/22:5)	0.10	0.40	TAG56:7-FA22:4	0.21	0.10	TAG50:2-FA18:0	0.20	0.11	TAG56:6-FA22:4	0.00	0.99

Abbreviations—FA: fatty acid; HEU: HIV-exposed uninfected; HUU: HIV-unexposed uninfected; IL: interleukin; hs-CRP: high-sensitivity C-reactive protein; IFN-γ: interferon gamma; LPC: lysophosphatidylcholine; LPE: lysophosphatidylethanolamine, LP-CE: lysophospholipid and cholesteryl ester; PE: phosphatidylethanolamine; PUFA: polyunsaturated triacylglycerol; TNFR: soluble TNF-α receptor; sCD14: soluble CD14; sCD163: soluble CD163; TNFα: tumor necrosis factor alpha; TAG: triacylglycerol, SM-TAG: saturated and monosaturated TAG. * *p* < 0.05; ** *p* < 0.01.

## Data Availability

Due to privacy protections and the relatively small sample size, the data are not available. However, collaborations are welcome with reasonable requests.

## Supplementary Materials

The following supporting information can be downloaded at https://www.mdpi.com/article/10.3390/metabo15120765/s1: Supplemental Table S1: The metabolites, Pearson R for the correlation of each metabolite with the eigenmodule, and *p*-value. Supplemental Table S2: The metabolites and their assigned module based on color.

## Abbreviations

The following abbreviations are used in this manuscript:

ART	antiretroviral therapy
HEU	HIV-exposed uninfected
HUU	HIV-unexposed uninfected
hs-CRP	High-sensitivity C-reactive protein
IFN-γ	interferon gamma
TNFα	tumor necrosis factor alpha
sTNFR	soluble(s) TNF-α receptor
IL	interleukin
sCD14	soluble CD14
sCD163	soluble CD163
PWH	people living with HIV
PWoH	people without HIV

## References

[1] Shiau S., Jacobson D.L., Huo Y., Kacanek D., Yee L.M., Williams D.B., Haddad L.B., Serghides L., Powis K., Sperling R.S. (2023). Unique Profile of Inflammation and Immune Activation in Pregnant People with HIV in the United States. J. Infect. Dis..

[2] Vyas P., Mathad J.S., Leu C.-S., Naik S., Alexander M., Araújo-Pereira M., Kulkarni V., Deshpande P., Yadana S., Andrade B.B. (2021). Impact of HIV Status on Systemic Inflammation During Pregnancy. AIDS.

[3] Hunt P.W., Lee S.A., Siedner M.J. (2016). Immunologic Biomarkers, Morbidity, and Mortality in Treated HIV Infection. J. Infect. Dis..

[4] Sandler N.G., Wand H., Roque A., Law M., Nason M.C., Nixon D.E., Pedersen C., Ruxrungtham K., Lewin S.R., Emery S. (2011). Plasma Levels of Soluble CD14 Independently Predict Mortality in HIV Infection. J. Infect. Dis..

[5] Shafiq M., Mathad J.S., Naik S., Alexander M., Yadana S., Araújo-Pereira M., Kulkarni V., Deshpande P., Kumar N.P., Babu S. (2021). Association of Maternal Inflammation During Pregnancy with Birth Outcomes and Infant Growth Among Women with or Without HIV in India. JAMA Netw. Open.

[6] Sevenoaks T., Wedderburn C.J., Donald K.A., Barnett W., Zar H.J., Stein D.J., Naudé P.J.W. (2021). Association of Maternal and Infant Inflammation with Neurodevelopment in HIV-Exposed Uninfected Children in a South African Birth Cohort. Brain Behav. Immun..

[7] Reikie B.A., Adams R.C.M., Leligdowicz A., Ho K., Naidoo S., Rusk C.E., de Beer C., Preiser W., Cotton M.F., Speert D.P. (2014). Altered Innate Immune Development in HIV-Exposed Uninfected Infants. J. Acquir. Immune Defic. Syndr..

[8] Jao J., Abrams E.J. (2014). Metabolic Complications of in Utero Maternal HIV and Antiretroviral Exposure in HIV-Exposed Infants. Pediatr. Infect. Dis. J..

[9] Jao J., Kirmse B., Yu C., Qiu Y., Powis K., Nshom E., Epie F., Tih P.M., Sperling R.S., Abrams E.J. (2015). Lower Preprandial Insulin and Altered Fuel Use in HIV/Antiretroviral-Exposed Infants in Cameroon. J. Clin. Endocrinol. Metab..

[10] Jao J., Bonner L.B., Dobinda K., Powis K.M., Sun S., Legbedze J., Mmasa K.N., Makhema J., Mmalane M., Kgole S. (2024). Lower Insulin Sensitivity Through 36 Months of Life with in Utero HIV and Antiretroviral Exposure in Botswana: Results from the Tshilo Dikotla Study. Clin. Infect. Dis..

[11] Hornburg D., Wu S., Moqri M., Zhou X., Contrepois K., Bararpour N., Traber G.M., Su B., Metwally A.A., Avina M. (2023). Dynamic Lipidome Alterations Associated with Human Health, Disease and Ageing. Nat. Metab..

[12] Schoeman J.C., Moutloatse G.P., Harms A.C., Vreeken R.J., Scherpbier H.J., Van Leeuwen L., Kuijpers T.W., Reinecke C.J., Berger R., Hankemeier T. (2017). Fetal Metabolic Stress Disrupts Immune Homeostasis and Induces Proinflammatory Responses in Human Immunodeficiency Virus Type 1- and Combination Antiretroviral Therapy-Exposed Infants. J. Infect. Dis..

[13] Jao J., Balmert L.C., Sun S., Qiu Y., Kraus T.A., Kirmse B., Sperling R.S., Abrams E.J., Myer L., Arpadi S. (2021). Distinct Cord Blood C-Peptide, Adipokine, and Lipidomic Signatures by in Utero HIV Exposure. Pediatr. Res..

[14] Olsen I.E., Groveman S.A., Lawson M.L., Clark R.H., Zemel B.S. (2010). New Intrauterine Growth Curves Based on United States Data. Pediatrics.

[15] Kuhn M., Jackson S., Cimentada J. *Corrr*: *Correlations in R 2025*; The Comprehensive R Archive Network (CRAN). https://github.com/tidymodels/corrr.

[16] Francis E.C., Kechris K., Johnson R.K., Rawal S., Pathmasiri W., Rushing B.R., Du X., Jansson T., Dabelea D., Sumner S.J. (2024). Maternal Serum Metabolomics in Mid-Pregnancy Identifies Lipid Pathways as a Key Link to Offspring Obesity in Early Childhood. Int. J. Mol. Sci..

[17] Langfelder P., Horvath S. (2008). WGCNA: An R Package for Weighted Correlation Network Analysis. BMC Bioinform..

[18] Perez De Souza L., Alseekh S., Brotman Y., Fernie A.R. (2020). Network-Based Strategies in Metabolomics Data Analysis and Interpretation: From Molecular Networking to Biological Interpretation. Expert. Rev. Proteom..

[19] Akoto C., Norris S.A., Hemelaar J. (2021). Maternal HIV Infection Is Associated with Distinct Systemic Cytokine Profiles Throughout Pregnancy in South African Women. Sci. Rep..

[20] Bebell L.M., Ngonzi J., Butler A., Kumbakumba E., Adong J., Loos C., Boatin A.A., Bassett I.V., Siedner M.J., Williams P.L. (2024). Distinct Cytokine Profiles in Late Pregnancy in Ugandan People with HIV. Sci. Rep..

[21] Lohman-Payne B., Koster J., Gabriel B., Chilengi R., Forman L.S., Heeren T., Duffy C.R., Herlihy J., Crimaldi S., Gill C. (2022). Persistent Immune Activation in Human Immunodeficiency Virus-Infected Pregnant Women Starting Combination Antiretroviral Therapy After Conception. J. Infect. Dis..

[22] Prendergast A.J., Rukobo S., Chasekwa B., Mutasa K., Ntozini R., Mbuya M.N.N., Jones A., Moulton L.H., Stoltzfus R.J., Humphrey J.H. (2014). Stunting Is Characterized by Chronic Inflammation in Zimbabwean Infants. PLoS ONE.

[23] Wilkinson A.L., Pedersen S.H., Urassa M., Michael D., Andreasen A., Todd J., Kinung’hi S.M., Changalucha J., McDermid J.M. (2017). Maternal Systemic or Cord Blood Inflammation Is Associated with Birth Anthropometry in a Tanzanian Prospective Cohort. Trop. Med. Int. Health.

[24] Grebenciucova E., VanHaerents S. (2023). Interleukin 6: At the Interface of Human Health and Disease. Front. Immunol..

[25] Dahlgren J., Samuelsson A.-M., Jansson T., Holmäng A. (2006). Interleukin-6 in the Maternal Circulation Reaches the Rat Fetus in Mid-Gestation. Pediatr. Res..

[26] Liu P., Zhu W., Chen C., Yan B., Zhu L., Chen X., Peng C. (2020). The Mechanisms of Lysophosphatidylcholine in the Development of Diseases. Life Sci..

[27] Rhee E.P., Cheng S., Larson M.G., Walford G.A., Lewis G.D., McCabe E., Yang E., Farrell L., Fox C.S., O’Donnell C.J. (2011). Lipid Profiling Identifies a Triacylglycerol Signature of Insulin Resistance and Improves Diabetes Prediction in Humans. J. Clin. Investig..

[28] Fessler M.B., Rudel L.L., Brown J.M. (2009). Toll-like Receptor Signaling Links Dietary Fatty Acids to the Metabolic Syndrome. Curr. Opin. Lipidol..

[29] Sengottuvel V., Hota M., Oh J., Galam D.L., Wong B.H., Wenk M.R., Ghosh S., Torta F., Silver D.L. (2023). Deficiency in the Omega-3 Lysolipid Transporter Mfsd2a Leads to Aberrant Oligodendrocyte Lineage Development and Hypomyelination. J. Clin. Investig..

[30] Wajant H., Siegmund D. (2019). TNFR1 and TNFR2 in the Control of the Life and Death Balance of Macrophages. Front. Cell Dev. Biol..

[31] Fiedler T., Fairless R., Pichi K., Fischer R., Richter F., Kontermann R.E., Pfizenmaier K., Diem R., Williams S.K. (2023). Co-Modulation of TNFR1 and TNFR2 in an Animal Model of Multiple Sclerosis. J. Neuroinflamm..

[32] Fischer R., Kontermann R.E., Pfizenmaier K. (2020). Selective Targeting of TNF Receptors as a Novel Therapeutic Approach. Front. Cell Dev. Biol..

[33] Francis E.C., Dumolt J.H., Zemski-Berry K., Jansson T., Powell T.L. (2025). Maternal Plasma Choline Levels Are Positively Correlated with Maternal and Placental Phospholipid-DHA Content in Females with Obesity Who Receive DHA Supplementation. J. Nutr..

[34] Yamamoto N., Hashimoto A., Takemoto Y., Okuyama H., Nomura M., Kitajima R., Togashi T., Tamai Y. (1988). Effect of the Dietary Alpha-Linolenate/Linoleate Balance on Lipid Compositions and Learning Ability of Rats. II. Discrimination Process, Extinction Process, and Glycolipid Compositions. J. Lipid Res..

[35] Jao J., Kacanek D., Yu W., Williams P.L., Patel K., Burchett S., Scott G., Abrams E.J., Sperling R.S., Van Dyke R.B. (2020). Neurodevelopment of HIV-Exposed Uninfected Infants Born to Women with Perinatally Acquired HIV in the United States. J. Acquir. Immune Defic. Syndr..

